# A Decade of Adolescent Pregnancy—Risk Assessment—A Tertiary Center Retrospective Analysis

**DOI:** 10.3390/diagnostics16111666

**Published:** 2026-05-28

**Authors:** Daniela Roxana Matasariu, Demetra Gabriela Socolov, Iuliana-Elena Bujor, Maria Elena Nita, Gabriel-Ioan Anton, Alexandra Ursache, Carmen Pintilescu, Monica Titianu, Vasile Lucian Boiculese, Ecaterina Tomaziu-Todosia Anton, Alexandru Carauleanu

**Affiliations:** 1 Grigore T. Popa University of Medicine and Pharmacy Iasi, 700115 Iasi, Romania; daniela.matasariu@umfiasi.ro (D.R.M.); demetra.socolov@umfiasi.ro (D.G.S.); nita.maria-elena@d.umfiasi.ro (M.E.N.); anton.gabriel-ioan@d.umfiasi.ro (G.-I.A.); alexandra.ursache@umfiasi.ro (A.U.); tomaziu-todosia.ecaterina@d.umfiasi.ro (E.T.-T.A.); ale.carauleanu@umfiasi.ro (A.C.); 2 Department of Obstetrics and Gynecology, Cuza Voda Hospital, 700038 Iasi, Romania; moni_titianu@yahoo.com; 3 Faculty of Economics and Business Administration, Alexandru Ioan Cuza University, 700505 Iasi, Romania; carmen.pintilescu@uaic.ro

**Keywords:** adolescent pregnancy, teenagers, obesity, overweight, pregnancy complications, large for gestational age fetuses (LGA)

## Abstract

**Background/Objectives:** Adolescent pregnancy, defined as pregnancy occurring between ages 10 and 19, remains a pressing global health concern with significant disparities in prevalence and outcomes across countries. Early and systematic diagnostic screening may allow timely risk stratification and adequate management. **Methods:** We conducted this retrospective cohort study at a tertiary referral center from January 2015 through December 2024, including all women who delivered live fetuses at our facility, analyzing adolescent pregnancy outcomes in our region and comparing them with adult pregnancy outcomes. **Results:** Younger adolescents have higher rates of vaginal infections (45.3% vs. 38.1%), chorioamnionitis, urinary tract infections (6% vs. 4.9%), preterm birth, higher cesarean section rates, SGA and FGR fetuses, with more frequent NICU admissions than older adolescents. Adolescent pregnancies more often resulted in vaginal births compared to adult pregnancies but also showed higher rates of operative vaginal delivery, episiotomy, perineal tears, vaginal tears, and cervical lacerations. Gestational diabetes and excessive gestational weight gain were overall less common in adolescents, but pre-pregnancy maternal obesity was significantly more prevalent in the older adolescent group than in the younger ones. Gestational hypertension was about twice as frequent in adult pregnancies, while HELLP syndrome was approximately six times more common in adults than in adolescents. **Conclusions:** In summary, adolescent pregnancy presents both potential biological advantages and notable disadvantages, with outcomes resulting from the complex interplay of biological immaturity and socioeconomic factors. These results highlight the critical importance of implementing comprehensive early diagnostic screening protocols and structured antenatal care to facilitate earlier identification and mitigation of modifiable risk factors to improve both maternal and fetal outcomes.

## 1. Introduction

Adolescence, as defined by the World Health Organization (WHO), is the transitional period between childhood and adulthood, covering the ages of 10–19 years and involving significant biological, psychological, and social changes [1,2]. This stage is further divided into early adolescence (10–14 years) and late adolescence (15–19 years) [2].

Adolescence is a critical transitional phase from childhood to adulthood, marked by substantial physiological, metabolic, and anatomical changes. Pregnancy during this period has been linked to adverse outcomes across health, social, and economic domains [3].

Adolescent pregnancy, defined as pregnancy occurring between the ages of 10 and 19 years old, remains a pressing global health concern with significant disparities in prevalence and outcomes across different countries. While most developed nations have experienced a decline in teenage pregnancy rates due to improved education, access to contraceptives, and social support [4,5], low- and middle-income countries still account for over 90% of adolescent births, with more than 16 million girls aged 15–19 and nearly 1 million under 15 giving birth each year [1,2,6]. The causes of AP’s complications are multifactorial—biological immaturity, poor socioeconomic conditions, inadequate healthcare access, and limited education all play significant roles [7,8,9,10].

Pregnancy during adolescence not only involves significant physiological risks but also carries deep psychological and social effects. Adolescents who become pregnant often face notable social stigma, judgment, and discrimination, which can intensify feelings of isolation, anxiety, and depression during an already vulnerable developmental stage [4,5,7,11,12].

On the maternal side, teenagers face increased risks for obstetric complications such as sexually transmitted diseases, high rates of abortion, premature birth, premature rupture of membranes (PROM), preterm premature rupture of membranes (PPROM), preeclampsia, eclampsia, anemia, postpartum hemorrhage, high incidence of instrumental delivery, tissue trauma (perineal, vaginal, and cervical lacerations), delivery complications, and maternal mortality [8,12,13,14,15,16,17,18,19,20,21,22,23]. These pregnancies often involve delayed prenatal care and poor nutritional status, both of which contribute to adverse maternal outcomes [24].

Overweight and obese women are more likely to experience pregnancy complications such as an increased risk of miscarriage, gestational diabetes, hypertension, preeclampsia, eclampsia, prolonged labor, more frequent inductions, higher rates of cesarean sections, and postpartum hemorrhage. Fetal outcomes are also negatively affected by preterm deliveries, post-term births, higher rates of macrosomia (newborns weighing over 4000 g), and large for gestational age (LGA) fetuses with birthweights exceeding the 90th percentile. Macrosomia and LGA fetuses have a consequently higher incidence of maternal and fetal obstetric trauma [25].

Neonatal complications are equally serious, with higher rates of preterm birth, low birth weight, delivery trauma, neonatal intensive care admission, and three times higher neonatal mortality compared to adult pregnancies [17,24,26]. Additionally, adolescent pregnancies often lead to inadequate gestational weight gain, impaired fetal growth, and poor Apgar scores at birth [21,27].

Despite extensive literature, there are conflicting results regarding the primary determinants, whether biological or social. Maternal and fetal outcomes continue to challenge public health strategies [21,28]. In Eastern Europe, particularly in Romania and Bulgaria, teenage pregnancy rates remain among the highest in the continent, further emphasizing the need for targeted research and intervention in these regions. However, when analyzing only European countries, Romania ranks second [9,29].

Although the national adolescent pregnancy rate has decreased from 11.79% in 2009 to 9.15% in 2021, it continues to be a significant public health concern, with higher rates of maternal and fetal morbidity and mortality, particularly among mothers under 15 years old [2,30].

Accumulating evidence shows that adolescent pregnancy is linked to adverse neonatal outcomes, highlighting the need for a study to develop effective strategies to improve understanding and minimize the impact of adolescent pregnancy in Romania. Despite well-documented adverse outcomes, few studies have evaluated possibilities of improvement offered by particularized protocols focused on reducing specific risks in adolescent pregnancies.

Adolescent pregnancies continue to strain the healthcare system, requiring ongoing and sustained efforts to lower both its occurrence and complications. Our study aimed to evaluate the AP rate, identify factors affecting pregnancy outcomes, and offer further insights into the challenges faced by AP.

## 2. Materials and Methods

### 2.1. Study Design

**Figure 1:**
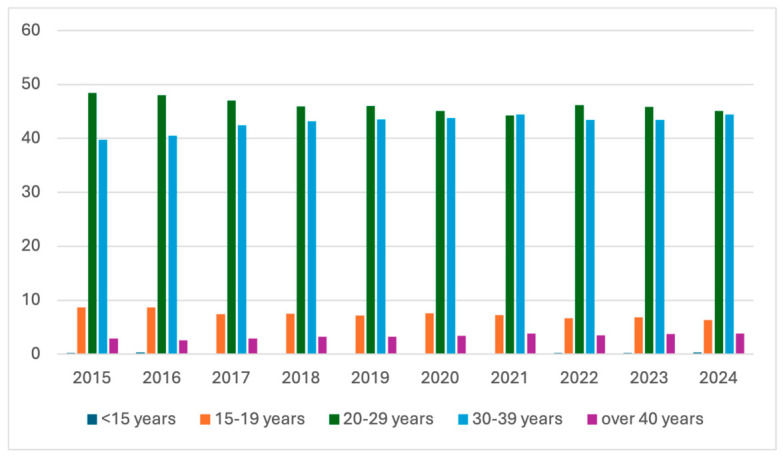
Distribution births depending on maternal age in the 10-year selected timeframe.

This single-center retrospective cohort study was conducted in a tertiary referral hospital from January 2015 through December 2024, involving all women who delivered live fetuses in Iasi, Romania (Figure 1). Sociodemographic characteristics were recorded for each mother, including age, place of birth, and pregnancy outcomes. For each newborn, we gathered sociodemographic data (sex), medical data (gestational age, birth weight), and immediate neonatal outcomes (Apgar score, admission to the neonatal intensive care unit, and evaluation of fetal malformations).

**Figure 2:**
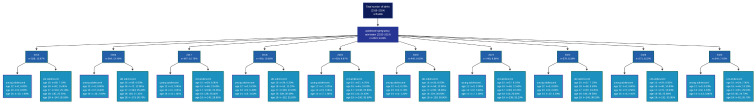
Flowchart with the distribution of adolescent pregnancies in the ten-year selected timeframe.

A flowchart showing the selection process for our adolescent cohort appears in Figure 2. We examined all data records from our institution related to underage adolescent mothers and their infants. The data were verified for accuracy and missing values by reviewing both the mother’s and the newborn’s medical records.

The gestational age of these pregnancies was determined by our hospital’s healthcare providers based on the time between the delivery date and the patient’s last menstrual period, adjusted using either first- or second-trimester morphological scans or the patient’s only ultrasound exam.

### 2.2. Inclusion Criteria

We included in our study all adolescents aged 12 to 19 who gave birth after 24 full weeks of gestation with a neonatal weight over 500 g during the 10-year period. All women who gave birth in the same timeframe, aged 20 to 24, were part of the control group.

### 2.3. Exclusion Criteria

Due to increased maternal–fetal complications related to age and higher use of assisted reproduction techniques, we excluded pregnant women over 24 years of age from our control group.

The data collection was carried out with the approval of the Ethics Committee of Cuza Voda Hospital in Iasi (2000/11 February 2025).

Maternal age was defined as the mother’s age in completed years at the time of delivery. We broadly categorized women into adults (20–24 years old, used as a reference group) and adolescents (<19 years). The adolescents were further divided into three groups: young adolescents under 15 years old, older adolescents between 15 and 18 years old, and the oldest adolescents aged 18 and 19 years.

We chose the 20–24-year-old group as the reference adult population because it reflects a relatively young reproductive cohort with fewer chronic maternal conditions, lower usage of assisted reproductive technologies like IVF, and fewer age-related obstetric risks compared to older groups. Additionally, this age range is biologically and reproductively close to adolescents, allowing for a more balanced comparison while reducing the confounding influence of advanced maternal age and its related health issues.

### 2.4. Outcomes

Preterm birth is defined as a birth occurring between 24 weeks and 36 weeks and 6 days of gestation. The WHO further classifies preterm births into three categories: extremely preterm (less than 28 weeks), very preterm (28 to less than 32 weeks), and moderate to late preterm (32 to 37 weeks) [21].

We also defined severe laceration at birth as a third- or fourth-degree laceration.

A low Apgar score was defined as less than 7 at 5 min [23].

All newborns who die within the first week after birth are called neonatal deaths [24].

Anemia was defined as a hemoglobin level below 11 mg/dL [26].

Maternal excessive fetal weight refers to a weight gain of over 16 kg during pregnancy. Maternal overweight is defined as a body mass index (BMI), calculated by dividing body weight in kilograms (kg) by height in meters squared, between 25 and 29.9. Maternal obesity is categorized into grades: grade I with a BMI between 30 and 34.9; grade II with a BMI between 35 and 39.9; and grade III with a BMI over 40 [31].

The vaginal infection group consisted of patients with positive vaginal and/or cervical cultures showing significant microbial growth (>3+++) linked to an inflammatory response. They delivered between 6 and 72 h after admission, depending on their clinical and laboratory status, which included factors such as fever, leukocytosis, and elevated CRP levels.

### 2.5. The Ultrasound Diagnostic Criteria

- Fetus according to gestational age (AGA)—fetuses with a birth weight between the 10th and 90th percentiles for the gestational age [27].
- Small for gestational age (SGA)—fetuses with a birth weight below the 10th percentile for gestational age but above the 3rd percentile, based on their genetic growth potential [28].
- Fetal growth restriction (FGR)—an estimated fetal weight at or below the 3rd percentile compared to normal fetal weight for the gestational age; early-onset FGR (<32 weeks of gestation) presents with an abdominal circumference/estimated fetal weight (AC/EFW) under the 3rd percentile or an absent end-diastolic flow in the umbilical artery (US-AEDF), while late-onset FGR (≥32 weeks of gestation) presents with an AC/EFW under the 3rd percentile or at least two of the following three criteria: AC/EFW below the 10th percentile, crossing centiles by more than 2 quartiles, and/or abnormal Doppler findings (uterine artery pulsatility index > 95th percentile, umbilical artery pulsatility index > 95th percentile, and/or cerebro-placental ratio < 5th percentile) [32].
- Large for gestational age (LGA)—birthweight above the 90th percentile for gestational age [29].

We reviewed the prenatal ultrasonographic growth curve in the patient’s medical records to differentiate moderate FGR from SGA. When medical records were unavailable because patients could not reach healthcare providers, we consulted our neonatologists.

The Apgar score and admission to the neonatal intensive care unit (NICU) were analyzed as perinatal outcomes. The evaluated peripartum outcomes included the prevalence of C-sections (cesarean sections) and their primary causes, the need to perform an episiotomy, the requirement for an operative vaginal delivery, and postpartum maternal complications.

The relationship between overweight and obesity among participants and their parity was studied. Participants were grouped into three categories: first-time mothers, second-time mothers, and those with their third or later child. The analysis examined how overweight and obesity are associated with infertility treatments, miscarriages (calculated as the difference between pregnancies and live births), pregnancy and perinatal complications such as gestational diabetes, eclampsia, pregnancy-induced hypertension, oligohydramnios, mode of delivery (C-section or vaginal birth), perineal injuries during delivery (perineal lacerations), and incomplete placenta expulsion. The study also investigated how women’s overweight and obesity influence newborn outcomes, including birth weight, birth length, APGAR score at one minute, occurrence of macrosomia, perinatal injuries, and breastfeeding challenges.

### 2.6. Statistical Analysis

The data analysis was conducted using the SPSS application, version 24 (IBM Corp. Released 2016. IBM SPSS Statistics for Windows, Version 24.0. IBM Corp.: Armonk, NY, USA).

For each type of variable individually, the statistics used to describe the data included absolute and relative frequencies or means and standard deviations. The frequencies of categorical variables were compared, and the average was considered for numerical data. In contingency tables, the Chi-square test was applied by default. Fisher’s exact test was used if the assumptions for the Chi-square test were not met.

The *t*-test or the Mann–Whitney test for two samples was used for continuous variables (ratio or interval scale).

A multinomial logistic regression model was estimated to assess the influence of predictors on the final outcome. The global significance of each predictor was evaluated using Likelihood Ratio Tests, and the model’s overall explanatory power was assessed using Pseudo R-square, specifically the Nagelkerke and Cox and Snell coefficients. The results of the estimated logistic regression model are presented in the table below.

## 3. Results

After applying the predefined inclusion and exclusion criteria, the study cohort consisted of a total of 4515 adolescent pregnancies over the selected 10-year period (Figure 1). No deliveries were recorded among 10- or 11-year-old adolescents in our institution. The annual distribution of these pregnancies, categorized by maternal age, is shown in the following flowchart (Figure 2), while Table 1 presents the socio-demographic characteristics, indicating that most originated from rural areas.

### 3.1. Maternal Outcomes in Adolescent Pregnancies

**Figure 3:**
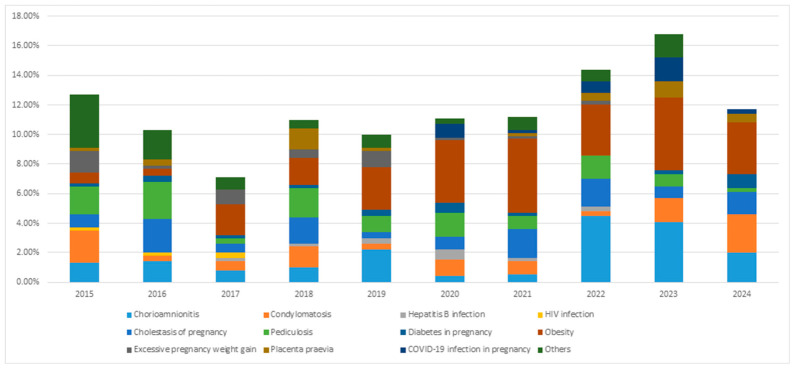
Other associated maternal pathologies.

The maternal characteristics and pregnancy-associated pathologies are summarized in both Table 2 and Figure 3. As expected, analysis of gestational age and parity revealed significant differences between the two adolescent subgroups. Of particular interest was the high proportion of multiparous cases found in older adolescents (15–19 years): 774 of 4398 were in their second pregnancy, and 100 were in their third or even fourth pregnancy (8/4398).

Although not reaching statistical significance, younger adolescents show higher rates of vaginal infections (45.3% vs. 38.1%) and urinary tract infections (6% vs. 4.9%) compared to older adolescents. Likewise, chorioamnionitis was more common in the younger group.

No differences were found among the three age groups (younger adolescents, older adolescents, and adults) in the incidence of intrahepatic cholestasis of pregnancy, hepatitis B, hepatitis C, HIV infection, or condylomatosis.

An increasing trend in twin pregnancies was observed with rising maternal age within the adolescent group. Gestational hypertension occurred at 2.6% in both adolescent subgroups, compared to 4.1% in adult pregnancies (no significant difference); preeclampsia rates were similar across all groups. No cases of HELLP syndrome (Hemolysis, Elevated liver enzymes, Low platelet count) were reported in younger adolescents, while 0.2% occurred in older adolescents, with about a sixfold higher incidence in adults.

Gestational diabetes and excessive weight gain during pregnancy were less common overall in adolescent pregnancies. However, maternal obesity before pregnancy was significantly more prevalent in the older adolescent group compared to the younger ones.

**Figure 4:**
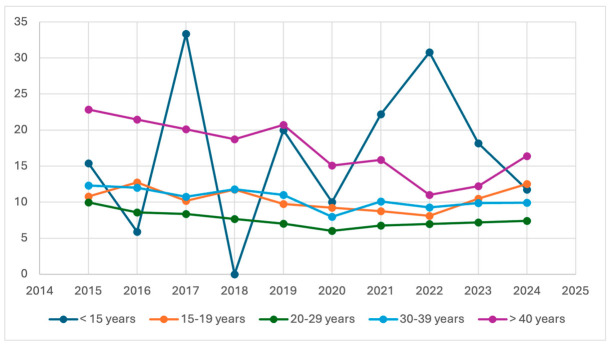
Trends in preterm birth depending on maternal age.

Regarding gestational age at delivery, preterm birth (under 37 weeks of gestation) was significantly more common among younger adolescents, 22.2% (26/117), compared to 12.1% (534/4398) in older ones (Figure 4). This difference was consistent across all preterm subcategories (<28 weeks of gestation—0.9% vs. 0.07%; 28–31 weeks of gestation—3.4% vs. 1.8%; 32 to 36 weeks of gestation—3.4% vs. 2.1%), with late preterm births contributing importantly to the overall disparity. Term births comprised 77.8% in younger adolescents versus 87.9% in older adolescents, with mean gestational ages at delivery of 37.62 weeks compared to 38.22 weeks, respectively.

Analysis of delivery modes revealed a higher cesarean section rate among younger adolescents (47%) compared to older adolescents (37.5%), with spontaneous vaginal births remaining predominant in both groups (53% vs. 62.5%). The difference in vaginal birth rates was statistically significant (*p* = 0.037). Indications for cesarean section did not differ significantly between subgroups. Operative vaginal delivery (vacuum/forceps-assisted) increased with maternal age, being less common in younger adolescents than in older ones or adult pregnancies.

The rates of episiotomy, perineal tears, vaginal tears, and cervical lacerations showed no significant differences between the two adolescent groups (*p*—0.432; 0.597; 0.767; and 0.083, respectively). Postpartum uterine curettage requirements were similar between adolescent subgroups but significantly higher in adolescents overall compared to adults (*p* < 0.05).

No significant differences were found in anemia incidence between the adolescents’ groups (*p*—0.052), antepartum/postpartum hemoglobin levels, or need for blood transfusion (*p*—0.215). Two cases of peripartum hysterectomy due to uterine atony occurred in the 15–19 years old group, with none in the younger subgroup.

### 3.2. Neonatal Outcomes in Adolescent Pregnancies

**Figure 5:**
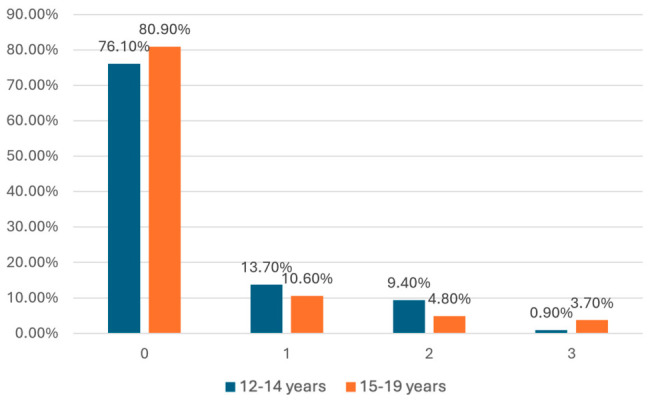
Neonatal weight outcome in adolescent pregnancies (AGA—0; SGA—1; FGR—2; LGA—3).

Table 3 provides an overview of perinatal characteristics. Fetal sex distribution showed no statistically significant differences between the two adolescent groups. However, mean birth weight was significantly lower in the younger adolescent group (12–14 years) by approximately 240 g compared with the older adolescent group (15–19 years) (*p* < 0.05). As anticipated, the younger subgroup exhibited significantly higher rates of SGA and FGR fetuses (*p* = 0.033), along with a significantly greater proportion of admission to the neonatal intensive care unit (NICU) (*p* = 0.031). Fetal weight outcomes in our adolescent pregnancy cohort are depicted in Table 3 and Figure 5. No significant differences were observed between the adolescent groups in the primary indications for NICU admission (primarily low birth weight LBW and respiratory distress syndrome; *p* = 0.630), or in Apgar scores at 1 and 5 min (Table 3). Conversely, LGA fetuses were significantly more common in the older adolescent group (*p* = 0.033) (Table 3).

**Figure 6:**
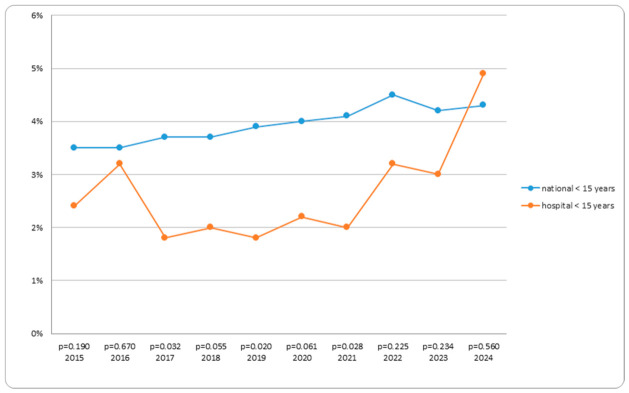
Our tertiary center young adolescent birth trend compared to our national statistic results.

**Figure 7:**
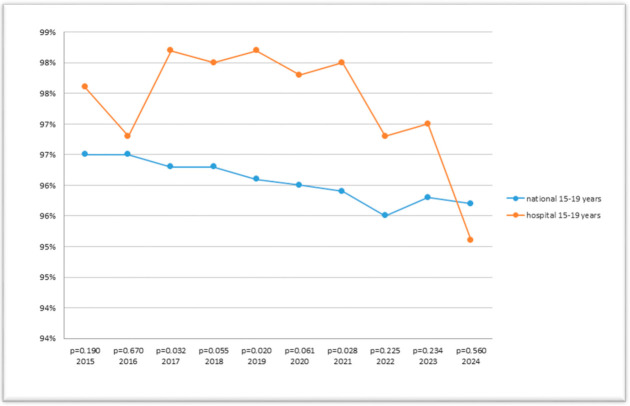
Our tertiary center older adolescent birth trend compared to our national statistic results.

The differences between adolescent births in our tertiary center compared to our available national statistics are illustrated in Figure 6 and Figure 7. Figure 6 presents our hospitals’ young adolescent trend compared to national statistics data. Our hospital registered fewer statistically significant 12- to 14-year-old adolescent births compared to national data in 2017, 2019, and 2021 (*p* values of 0.032, 0.02 and 0.028 respectively), with the year 2024 marking a significant increase compared to national statistics (Figure 6). Concerning the incidence of older adolescent births, our tertiary facility surpasses the registrations from national statistics, with a significant nadir in 2024 (Figure 7).

### 3.3. Adolescent Pregnancy Outcomes Compared to Adult Pregnancies

Compared to the adult group (women aged over 20 years at delivery), preterm birth rates were significantly lower in adults, consistent with a gradual decline in vaginal infections as maternal age increases. Urinary tract infection rates were similar between younger adolescents and adults but were significantly lower in older adolescents compared to adults (*p* < 0.01). The incidence of chorioamnionitis was comparable between the younger adolescent group and adults.

Gestational hypertension occurred roughly twice as often in adult pregnancies (4.1% vs. 2.6% in adolescents), although preeclampsia rates showed no significant differences between groups. HELLP syndrome was about six times more common in adults than in adolescents.

Gestational diabetes was significantly more common in adult pregnancies (*p* < 0.01). Likewise, both pre-pregnancy maternal obesity and excessive gestational weight gain were statistically significantly more frequent in adults compared with adolescents overall (*p* < 0.01). No differences were observed among the three groups (younger adolescents, older adolescents, adult pregnancies) regarding the incidence of intrahepatic cholestasis of pregnancy. However, adult pregnancies showed significantly higher rates of hepatitis B, human immunodeficiency virus (HIV), and condylomatosis (*p* < 0.05).

The mode of delivery varied significantly between adolescent and adult pregnancies. A considerably higher percentage of adolescents achieved successful vaginal births (*p* < 0.01), compared to adult pregnant women, with statistically more vaginal births in the older adolescent group (*p* = 0.037). Births among adults required significantly fewer episiotomies and had lower rates of perineal, vaginal, and cervical lacerations (*p* < 0.05).

Both young adolescents and adult pregnancies showed similar percentages of anemia, but the difference was statistically significant when comparing the older adolescent group with adult women. Moreover, a higher percentage of adult women underwent peripartum hysterectomy than adolescents, with 118 cases compared to two cases (*p* = 0.014).

Neonatal outcomes further emphasized age-related trends: the proportion of AGA fetuses increased with maternal age, while SGA and FGR rates were lowest in adults, nearing those seen in older adolescents. LGA rates in adults were similar to those in older adolescents. Fetal sex distribution showed no significant differences between adolescent and adult groups, but the average birth weight was still significantly lower in adolescent pregnancies overall (by 105 g; *p* < 0.05). NICU admission rates were significantly lower in adult pregnancies compared to adolescents (*p* = 0.007), although Apgar scores did not differ significantly across groups.

A multinomial logistic regression model was estimated to assess the influence of predictors on the final outcome. For the dependent variable, the AGA category was the reference group. The predictors included in the model were: pregnant women’s age, residence area (with the urban area as the reference category), gestational age (with over 37 weeks of gestation as the reference category), anemia, primiparity, cesarean section, operative vaginal delivery, vaginal and urinary infections (with the “yes” category as the reference category), and pregnancy hypertensive disorders (with the “yes” category as the reference category). The results of the estimated logistic regression model are presented in Table 4 below. Due to the small number of cases, the variables NICU admission (either due to small birth weight or respiratory distress), excessive weight gain, obesity, urinary infection, and multiple pregnancy could not be included in the multinomial regression model.

The estimations obtained shows that maternal age (*p* value less than 0.001), gestational age (*p* value less than 0.001), residency (*p* value 0.021) and maternal anemia (*p* value 0.025) are the most important predictors for the dependent variable.

For the category SGA maternal age, gestational age, residency and maternal anemia are the most important predictors for the dependent variable. Maternal age increases the risk of SGA with 17.7% (β = 0.163 OR = 1.177). Gestational age is also an important predictor for SGA; the risk of having an SGA child is 68.9% lower in premature births than in term births (β = −1.166, OR = 0.312). Concerning anemia, pregnant women with moderate and severe anemia, with hemoglobin value less than 9.5 mg/dL, are 30.7% more likely to have SGA births than those without this associated pathology. Additionally, residence area (*p* = 0.021) has an influence for the dependent variable. Rural mothers have 68.9% lower chances of delivering an SGA baby compared to urban ones, but with a visible trend towards a higher risk for LGA fetuses, although not reaching statistical significancy.

## 4. Discussion

Adolescent pregnancy remains a major public health challenge worldwide, with profound implications for maternal, fetal, and neonatal health outcomes. Extensive literature has consistently examined the association between teenage pregnancy and adverse obstetric, perinatal, and neonatal consequences, highlighting considerable variability across diverse populations and healthcare settings [4,5,33].

Data from 2013 to 2021 show consistently high adolescent pregnancy rates in regions such as French Guiana, Latin America, the Caribbean, and Eastern Europe, including Romania [33,34,35]. While the global average adolescent pregnancy rate is about 11%, the rate in this study was 8.27%, which is below the international average [29,36,37], and aligns more closely with findings reported by Ranjbar et al. in 2023 [38]. Consistent with existing evidence, our results highlight a disproportionate number of adolescent pregnancies in rural areas, mainly due to limited educational opportunities and restricted access to healthcare [21,39]. This rural dominance reflects well-established patterns in Romania, where adolescent pregnancies are more common in rural and socioeconomically disadvantaged communities, as documented in national and regional reports [40,41,42].

Numerous studies have documented a significantly increased risk of preterm birth among adolescent mothers [36,42,43,44]. Brosens et al. have suggested that this elevated risk results from uterine immaturity and progesterone resistance, emphasizing a multifactorial cause [45]. Besides these biological factors, socioeconomic determinants such as limited education, high-risk sexual behaviors, higher anemia rates during pregnancy, increased occurrences of vaginal and urinary tract infections, inadequate prenatal care due to limited healthcare access, nutritional deficiencies, and substance abuse further contribute to adverse outcomes [46]. Although most evidence supports higher preterm delivery rates in adolescent pregnancies, only limited data indicate no significant difference compared to adult pregnancies. Our findings align with the majority of existing literature [47,48].

Smoking prevalence is relatively low, with 4.3% smokers in the younger adolescent group and 5.5% in the older one. An additional social concern is related to postpartum discharge patterns: although most mothers and infants were discharged home, a significant portion required placement in shelters, professional foster care, or, in some cases, led to hospital abandonment of the newborn.

Our data show a clear downward trend in preterm birth rates with increasing maternal age, with significantly fewer preterm deliveries in adult pregnancies. Preterm birth occurred in 22.2% of younger adolescent pregnancies, 12.1% of older adolescent pregnancies, and 9.2% of adult pregnancies (*p* > 0.01), consistent with previous reports [48]. International data reveal a characteristic “U”-shaped distribution of preterm birth, with increases at both ends of maternal age—adolescence and advanced age [49]. Similar to the findings by Cagli et al., the relatively high preterm rates in our adolescent group and lower rates in adults may reflect the tertiary-care nature of our institution, which manages high-risk pregnancies referred from peripheral facilities, including cases involving young adolescents [36]. The higher rate of vaginal infections in our adolescent population likely contributes to some of the increased preterm birth risk [46], with the decreasing trend in prematurity mirroring the decline in vaginal infection rates with age. These findings differ from some studies that report comparable preterm birth rates between adolescents and adults, often due to referral bias in tertiary centers that favor high-risk cases [49,50,51].

Notably, the rates of urinary tract infection and chorioamnionitis were comparable between younger adolescents and adults but significantly lower in older adolescents. These findings contrast with much of the existing literature, which often reports higher rates of these infections in adolescent mothers [52,53] or similar rates compared to adults [54,55], as well as comparable chorioamnionitis risk in adolescents relative to the general population [38]. Our urinary tract infection rate aligns with the 8% cited in the American Journal of Obstetrics and Gynecology (AJOG) consensus guidelines [56], whereas chorioamnionitis rates vary across broader estimates [57,58]. These differences likely stem from a combination of institutional protocols (including vaginal swab collection during the first trimester, at initial admission, and at 35–37 weeks of gestation, along with urine cultures in each trimester), as well as population-specific factors and potential selection bias. Importantly, only culture-positive cases were included for both infection types, which may further affect the reported incidences.

Elevated pre-pregnancy body mass index (BMI) and excessive gestational weight gain (GWG) are well-established risk factors for adverse maternal and neonatal outcomes, with global rates continuing to rise in recent decades [59]. Both factors are linked to increased rates of large-for-gestational-age (LGA) fetuses and higher cesarean section rates [60]. Teenage mothers tend to have a lower susceptibility to these conditions compared with adults [36]; however, pregnancy during late adolescence, when somatic growth is still ongoing, often leads to significant weight gain and central fat accumulation. Adolescents frequently experience greater GWG and postpartum weight retention than adults [61]. As Gigante et al. described, primiparous adolescents tend to gain more weight without a proportionate increase in height, whereas multiparous adolescents gain similar weight but significantly less height, resulting in BMI increases dependent on pregnancy duration. Unlike adults, who generally enter a phase of fat reduction after 28 weeks of gestation, growing adolescents continue to accumulate subcutaneous fat, which worsens obesity risk by limiting final height and increasing weight gain [61]. Both underweight statuses, common among adolescents, and overweight/obesity carry risks: underweight increases the likelihood of preterm birth and small-for-gestational-age (SGA) infants, while overweight and obesity raise the risk of gestational diabetes, hypertensive disorders, and LGA fetuses [62]. Gestational diabetes mellitus (GDM) is typically less common in adolescent pregnancies than in adult ones, a pattern that our study confirms, showing a significantly lower incidence among adolescents (*p* < 0. 01). This may be due to lower pre-pregnancy BMI serving as a protective factor, although it is also a risk factor for prematurity and SGA [36]. The prevalence of preeclampsia was similar between adolescent and adult pregnancies in our cohort, consistent with several other reports [54,62], but contrasting with studies that report significant differences, either lower [29] or higher [63,64], likely reflecting variations in ethnicity, geography, and sociodemographic factors [29,54,62,63,64,65].

Intrahepatic cholestasis of pregnancy occurred at similar rates in both adolescent and adult groups, while sexually transmitted infections like hepatitis B and HIV were more common in adult pregnancies. Conversely, condylomatosis was more prevalent among adolescents.

Cesarean section rates were 37.78% among adolescents overall and 60.2% in adult pregnancies. Similar patterns have been observed in Turkey, where adolescents show higher vaginal delivery rates [66], with cesarean rates increasing with maternal age in women aged 14–30 years. The higher likelihood of vaginal delivery in younger adolescents may be related to increased connective tissue elasticity [66]. Although our adult cesarean rate (60.2%), observed at a tertiary center managing complex cases, exceeds the World Health Organization’s recommended threshold, it reflects the global upward trend and surpasses rates in countries with high cesarean rates such as the Dominican Republic or Brazil [67]. Operative vaginal birth rates in our adolescent group were significantly higher than in adults, with a rising trend from younger to older adolescents, although still considerably lower than the 12% reported by Robillard et al. for 14–15-year-olds, compared to our 5.1% in the younger adolescent group and 3.6% in older adolescents [68]. These findings differ from those of Staniczek et al. and Khudor et al. [46,69]. While literature highlights biological advantages of nulliparity and younger age, such as stronger uterine contractility, efficient labor progression, and greater pelvic adaptability before skeletal maturity [46,68,69], our higher operative vaginal delivery rates may stem from institutional practices favoring vacuum extraction to avoid prolonged second-stage labor, a higher proportion of AGA fetuses (76.1%) in younger adolescents, lower cesarean rates in this subgroup, and referral bias typical for a tertiary facility managing complex cases.

Episiotomy rates and incidence of perineal, vaginal, and cervical lacerations were substantially higher in our adolescent cohort, consistent with Karaca et al.’s findings [70]. Our vaginal and cervical laceration rates of around 6% align with literature, but the perineal laceration incidence of 15.5% exceeded typical reports, likely secondary to elevated operative vaginal birth rates [70,71,72]. These rates declined markedly in adult deliveries. Younger adolescents often exhibit poorer collaboration during labor, contributing to higher operative vaginal delivery and laceration rate.

Anemia prevalence in our cohort was alarmingly high, affecting nearly half of all women delivering at our institution, with higher rates among both younger adolescents and adults. Although its prevalence did not reach statistical significance (*p* = 0.22), it remained more common in younger adolescents, likely due to increased nutritional demands needed to support both maternal growth and fetal development. Our estimates are comparable to reports from other middle-income countries but are twice the incidence reported in high-income settings. Anemia is associated with higher risks of preterm delivery, SGA and low-birth-weight neonates, Apgar score issues, and NICU admissions [36,37,46,63].

Although several studies associate adolescent pregnancy with a higher risk of low birth weight [5,45,72], results may be affected by the inclusion of very young adolescents aged 10 to 14 years. Conversely, some research reports no significant differences in SGA, low birth weight, preterm delivery, or fetal mortality [54,73,74]. Our study, however, found significantly increased risks of SGA and FGR among infants born to adolescent mothers. Similar findings by Ozdemirci et al. and Kartasli et al. suggest that higher rates of preterm delivery partly explain increased low birth weight [75,76]. Biological mechanisms, such as immature uterine and cervical vasculature and progesterone resistance, likely play a role [54,74,75,76]. Quinlivan et al. demonstrated that comprehensive prenatal care significantly reduces preterm birth risk in adolescents (OR 0.40, 95% CI: 0.25–0.62) [77]. Neonates of adolescent mothers face higher perinatal morbidity and mortality, with more frequent low 5-min Apgar scores (<7), consistent with most literature [77]. Elevated NICU admission rates in this group are supported by Kirbas et al. [77,78], although some studies report similar rates [38].

The causes of adverse maternal and neonatal outcomes in adolescent pregnancy are complex and should not be attributed solely to biological immaturity. Although younger maternal age may increase certain obstetric risks, factors such as socioeconomic status, nutrition, access to prenatal care, education, and behavioral factors likely play a significant role in pregnancy outcomes. Recent research highlights the need to view adolescent pregnancy outcomes within a broader biopsychosocial context rather than assuming they stem directly from biology [79]. Our multinomial logistic regression model revealed that rural residence appears to have a protective effect on the risk of having an SGA fetus, with a non-statistically significant trend toward higher risk for LGA fetuses. This pattern may reflect associations with better nutritional quality and/or different dietary patterns; greater physical activity and perhaps a less sedentary lifestyle; less maternal stress; lower air pollution; or even selection bias. Older mothers have a higher risk of SGA fetuses than adolescents, due to increased comorbidities, placental aging, and reduced uterine blood flow with less compliant uterine arteries. Many cases of FGR are late-onset, with frequent misclassification as SGA fetuses, thus increasing the SGA risk with advanced gestational age. Preterm birth often occurs before growth restriction fully manifests, with many preterm babies being AGA fetuses. In agreement with the literature, maternal anemia also increases the risk of SGA fetuses. Although the pseudo R-square values were modest (Cox and Snell R^2^ = 0.046, Nagelkerke R^2^ = 0.063), they indicate that the model accounts for a meaningful portion of the variance in outcome and are consistent with findings in multifactorial clinical studies where outcomes are influenced by a wide array of biological and environmental variables [80,81,82].

Consequently, the findings in our study should be interpreted carefully, and no definitive causal relationship can be drawn due to the retrospective nature of the study.

### Limitations

The present study has several limitations that should be acknowledged.

Using a single young adult reference group (20–24 years) may limit the generalizability of comparisons to the broader adult obstetric population. We recognize that choosing women aged 20–24 strictly as the adult reference group could lead to selection bias and might not fully reflect the broader adult obstetric population. Nonetheless, this age group was deliberately selected because it exemplifies a relatively healthy young reproductive demographic with fewer chronic comorbidities, less reliance on assisted reproductive technologies, and fewer age-related obstetric issues. Additionally, its biological closeness to adolescents facilitates a more balanced comparison and reduces confounding factors linked to advanced maternal age. Another limitation is the small number of young adolescent pregnancies included in our study. The limited number may impact the results we have obtained, and thus careful interpretation is needed.

As a retrospective cohort analysis conducted in a single tertiary center, the findings are inherently region-specific and reflect patterns observed in our country; therefore, our results cannot be completely extrapolated to national trends or other geographic and socio-economic settings. Although this center assures healthcare for a substantial proportion of the regional population, our single-site research restricts external validity, and multicenter investigations incorporating diverse Romanian contexts would be required to enhance generalizability and permit meaningful inter-regional comparisons. One also needs to consider selection bias, the risk of misclassification, and incomplete documentation when analyzing our retrospective collected data and their results. There were also other determinants of adverse maternal and fetal outcomes that weren’t documented in our research such as psychosocial stress, marital status and partner’s violence, nutritional profile, and education level. Similarly, information on pregnancy evolution, particularly serial hemoglobin levels and other dynamic antenatal parameters, was lacking, limiting deeper insight into longitudinal risk evolution.

The findings of this study should be interpreted in light of several limitations, including its single-center design at a tertiary referral center, which may introduce referral bias and limit the generalizability of the results to broader populations.

The strength of our research lies in the number of cases included, which allows for a broader view of this challenging healthcare issue. Additionally, the extended timeframe enabled us to observe the evolution and trends of potential pathologies. Our future approach aims to strengthen the evidence base with more precise risk stratification and targeted preventive interventions in this vulnerable population subgroup, with expanded multicenter research including as many determinants as possible to improve the accuracy of our results.

## 5. Conclusions

In summary, adolescent pregnancy entails both potential biologic advantages (lower rates of cesarean delivery, low incidence of gestational diabetes, overweight, obesity and increased gestational weight gain) and substantial disadvantages (increased frequency of vaginal infections, and premature birth; more frequent operative vaginal delivery; increased incidence of perineal, cervical and vaginal lacerations; higher SGA and FGR fetuses; increased NICU admission). These outcomes arise from the intricated interplay of biologic immaturity and socioeconomic determinants. While some studies indicate comparable outcomes between adolescent and adult pregnancies, the preponderance of evidence, including the present investigation, demonstrates significantly elevated risks in adolescent pregnancies, particularly in resource-limited settings. Comprehensive antenatal care represents a pivotal strategy for attenuating adverse outcomes in this vulnerable population. Implementation of targeted diagnostic protocols, combined with intensive antenatal education, could substantially improve outcomes in this high-risk group. The distinctive characteristics of adolescent pregnancies in our region revealed a higher frequency of vaginal infections, which could be substantially amended through improved antenatal care attendance, enhanced emotional and socio-economic support, and systematic period vaginal sampling. Early detection and treatment of such infections, as also detected in our work, may also contribute to lowering the incidence of preterm birth. Preterm delivery could be further prevented by proactive screening and management of vaginal and urinary tract infections, combined with serial cervical length evaluation and individualized therapeutic interventions. Additionally, the increased rate of operative vaginal deliveries observed in this population was associated with a higher incidence of perineal, cervical, and vaginal lacerations. These complications could be reduced by implementing targeted antenatal education programs that instruct adolescent mothers on pain management techniques, effective pushing during the second stage of labor, and strategies to minimize perineal trauma. Finally, the elevated occurrence of SGA and FGR cases might be positively influenced by enhanced antenatal surveillance, with prophylaxis measures, optimized nutritional counseling, and timely supplementation when indicated, ultimately leading to improved neonatal outcomes and potential reduction in NICU admission.

## Figures and Tables

**Table 1:** Demographic characteristics of the adolescent pregnancies included in our cohort.

Characteristics	12–14 Years	15–19 Years	*p*-Value
Total	117 (2.6%)	4398 (97.4%)	<0.01
Area			
Urban	45 (38.5%)	798 (18.1%)	<0.01
Rural	72 (61.5%)	3600 (81.9%)	
Discharged			
Home	106 (90.6%)	4268 (97.0%)	<0.01
Mother and baby shelter	7 (6.0%)	74 (1.7%)	
Professional foster care	3 (2.6%)	31 (0.7%)	
Hospital abandoned child	1 (0.9%)	25 (0.6%)	
Smoking habits			
Yes	5 (4.3%)	241 (5.5%)	0.57
No	112 (95.7%)	4156 (94.5%)	

**Table 2:** Maternal-associated pathologies in the selected ten-year timeframe.

Characteristics	12–14 Years	15–19 Years	>19 Years	*p*-Value I. 12–14 vs. >15–19 Years II. 15–19 vs. ≥20 Years III. 10–14 vs. ≥20 Years		
				I	II	III
Total	117 (0.2%)	4398 (7.5%)	54,496 (92.3%)			
Preterm birth						
Yes	26 (22.2%)	534 (12.1%)	4997 (9.2%)	<0.01	<0.01	<0.01
No	91 (77.8%)	3864 (87.9%)	49,499 (90.8%)			
Vaginal infections						
Yes	53 (45.3%)	1674 (38.1%)	14,600 (26.8%)	0.11	<0.01	<0.01
No	64 (54.7%)	2724 (61.9%)	39,896 (73.2%)			
Urinary tract infections						
Yes	7 (6%)	216 (4.9%)	4059 (7.4%)	0.59	<0.01	0.67
No	110 (94%)	4182 (95.1%)	50,437 (92.6%)			
Chorioamnionitis						
Yes	3 (2.6%)	74 (1.7%)	1398 (2.6%)	0.45	<0.01	0.99
No	114 (97.4%)	4324 (98.3%)	53,098 (97.4%)			
Twin pregnancy						
Yes	5 (4.3%)	39 (0.9%)	1738 (3.2%)	<0.01	<0.01	0.43
No	112 (95.7%)	4359 (99.1%)	52,758 (96.8%)			
Gestational hypertension						
Yes	3 (2.6%)	116 (2.6%)	2249 (4.1%)	0.99 F	<0.01	0.63 F
No	114 (97.4%)	4282 (97.4%)	52,247 (95.9%)			
Preeclampsia						
Yes	0 (0%)	40 (0.9%)	558 (1%)	0.62 F	0.51	0.63 F
No	117(100%)	4358 (99.1%)	53,938 (99%)			
HELLP syndrome						
Yes	0 (0%)	10 (0.2%)	696 (1.3%)	0.99 F	<0.01	0.41 F
No	117(100%)	4388 (99.8%)	53,800 (98.7%)			
Gestational diabetes						
Yes	0 (0%)	17 (0.4%)	1805 (3.3%)	0.99 F	<0.01	0.035 F
No	117(100%)	4381 (99.6%)	52,691 (96.7)			
Excessive gestational weight gain						
Yes	0 (0%)	28 (0.6%)	793 (1.5%)	0.99 F	<0.01	0.42 F
No	117(100%)	4370 (99.4%)	53,703 (98.5%)			
Maternal obesity						
Yes	0 (0%)	133 (3%)	2524 (4.6%)	0.05 F	<0.01	0.03
No	117(100%)	4265 (97%)	51,972 (95.4%)			
No	117(100%)	4397 (99.98%)	54,419 (99.9%)			
The gestational age at birth (weeks of gestation)						
extremely preterm (<28 weeks)	1 (0.9%)	30 (0.7%)	312 (0.6%)	0.012	0.011	<0.01
very preterm (28–31 weeks)	4 (3.4%)	79 (1.8%)	738 (1.4%)			
moderate to late preterm (32–36 weeks)	21 (17.9%)	425 (9.7%)	4758 (8.8%)			
term (≥37 weeks)	91 (77.8%)	3864 (87.9%)	48,549 (89.3%)			
Type of delivery						
Vaginal birth	62 (53.0%)	2747 (62.5%)	21,691 (39.8%)	0.037	<0.01	<0.01
C-section birth	55 (47.0%)	1651 (37.5%)	32,805 (60.2%)			
Operative vaginal birth (forceps/vacuum extraction)						
Yes	6 (5.1%)	159 (3.6%)	782 (1.4%)	0.32 F	<0.01	<0.01 F
No	111 (94.9%)	4239 (96.4%)	53,714 (98.6%)			
Maternal anemia (Hg < 11mg/dL)						
Yes	60 (51.3%)	1858 (42.3%)	31,020 (56.9%)	0.052	<0.01	0.22
No	57 (48.7%)	2538 (57.7%)	23,476 (43.1%)			
Episiotomy						
yes	50 (42.7%)	2041 (46.4%)	3596 (6.6%)	0.432	<0.01	<0.01
no	67 (57.3%)	2357 (53.6%)	50,900 (93.4%)			
Perineal laceration						
yes	16 (13.7%)	680 (15.5%)	6387 (11.7%)	0.597	<0.01	0.51
no	101 (86.3%)	3718 (84.5%)	48,109 (88.3%)			
Vaginal laceration						
yes	6 (5.1%)	254 (5.8%)	879 (1.6%)	0.767	<0.01	0.01 F
no	111 (94.9%)	4144 (94.2%)	53,617 (98.4%)			
Cervical laceration						
yes	7 (6%)	486 (11.1%)	1843 (3.4%)	0.083	<0.01	0.12 F
no	110 (94.0%)	3912 (88.9%)	52,653 (96.6%)			
Instrumental curettage						
yes	20 (17.1%)	548 (12.5%)	879 (1.6%)	0.136	<0.01	<0.01 F
no	97 (82.9%)	3850 (87.5%)	53,617 (98.4%)			
Hemostasis hysterectomy						
yes	0 (0.0%)	2 (0.05%)	118 (0.2%)	0.99 F	0.02	0.99 F
no	117 (100.0%)	4396 (99.95%)	54,378 (99.8%)			

HELLP syndrome—Hemolysis, Elevated liver enzymes, Low platelet count. F—Fisher’s exact test.

**Table 3:** Neonatal characteristics and perinatal outcomes.

Characteristics	12–14 Years	15–19 Years	≥20 Years	*p*-Value I. 12–14 vs. ≥20 Years II. 15–19 vs. ≥20 Years III. 12–14 ani vs. 15–19 Years		
				I	II	III
Birth weight (g) (Mann–Whitney Test)	2881.8 ± 591.7	3122.5 ± 569.1	3223.5 ± 582.5	<0.01	<0.01	<0.01
AGA (according to gestational age)	89 (76.1%)	3558 (80.9%)	48,978 (89.9%)	0.033	<0.01	<0.01
SGA (small for gestational age)	16 (13.7%)	465 (10.6%)	2848 (5.2%)			
FGR (fetal growth restriction)	11 (9.4%)	212 (4.8%)	800 (1.5%)			
LGA (large for gestational age)	1 (0.9%)	163 (3.7%)	1870 (3.4%)			
Fetal malformations						
No	114 (97.4%)	4301 (98%)	54,242 (99.5%)	0.73 F	<0.01	0.02 F
Yes	3 (2.6%)	90 (2%)	254 (0.5%)			
NICU admission						
yes	21 (17.9%)	505 (11.5%)	4004 (7.3%)	<0.01	<0.01	0.031
no	96 (82.1%)	3893 (88.5%)	50,492 (92.7%)			
Motivation for NICU admission						
respiratory distress	9 (42.9%)	243 (48.2%)	2826 (62.67%)	0.061	<0.01	0.630
low birth weight	12 (57.1%)	261 (51.8%)	1683 (37.3%)			
Neonatal sex						
masculine	53 (45.3%)	2268 (51.6%)	27,874 (51.1%)	0.181	0.60	0.24
feminine	64 (54.7%)	2130 (48.4%)	26,622 (48.9%)			

NICU—neonatal intensive care unit.

**Table 4:** The multinomial logistic regression model on final outcome.

Dependent Variable	Predictors	Coefficients	OR	95 * CI	*p*-Value
SGA	Constant	−3.775 (0.832)			0
	Age	0.163 (0.040)	1.177	[1.087–1.273]	0
	Residence area	−0.280 (0.122)	0.756	[0.595–0.959]	0.021
	Gestational age	−1.166 (0.120)	0.312	[0.246–0.394]	0
	Anemia	−0.237 (0.105)	0.789	[0.642–0.970]	0.025
	Vaginal infection	0.048 (0.104)	1.049	[0.855–1.287]	0.647
	Pregnancy hypertensive disorders	−0.390 (0.232)	0.677	[0.429, 1.068]	0.094
	Operative vaginal delivery	0.268 (0.301)	1.307	[0.725–2.356]	0.374
	Cesarean section birth	−0.20 (0.106)	0.980	[0.796–1.207]	0.849
	Parity	0.234 (0.131))	1.265	[0.979–1.635]	0.074
FGR	Constant	2.576 (1.015)			0.011
	Age	−0.273 (0.047)	0.761	[0.695–0.834]	0
	Residence area	−0.228 (0.166)	0.796	[0.575–1.102]	0.169
	Gestational age	−0.193 (0.200)	0.824	[0.557, 1.220]	0.334
	Anemia	−0.414 (0.148)	0.661	[0.495–0.883]	0.005
	Vaginal infection	−0.325 (0.139)	0.722	[0.550–0.948]	0.019
	Pregnancy hypertensive disorders	−0.632 (0.282)	0.531	[0.306–0.923]	0.025
	Operative vaginal delivery	0.466 (0.468)	1.594	[0.637–3.989]	0.319
	Cesarean section	−0.384 (0.141)	0.681	[0.516–0.899]	0.007
	Parity	0.512 (0.222)	1.669	[1.080–2.578]	0.021
LGA	Constant	−4.550 (1.323)			0.001
	Age	0.052 (0.062)	1.054	[0.934–1.189]	0.396
	Residence area	0.453 (0.235)	1.573	[0.992–2.493]	0.054
	Gestational age	0.544 (0.319)	1.723	[0.922–3.222]	0.088
	Anemia	0.729 (0.162)	2.074	[1.510–2.848]	0
	Vaginal infection	0.107 (0.166)	1.113	[0.804–1.540]	0.52
	Pregnancy hypertensive disorders	0.253 (0.468)	1.288	[0.515–3.221]	0.588
	Operative vaginal delivery	−0.637 (0.386)	0.529	[0.248–1.128]	0.099
	Cesarean section	−0.892 (0.166)	0.410	[0.296–0.567]	0
	Parity	0.095 (0.205)	1.099	[0.735–1.643]	0.645

* The reference category for the dependent variable is the category normal weight. For the independent variable, the residence area, the reference category is the urban area; for gestational age, the reference category is the category under 37 weeks of gestation; for anemia, vaginal and urinary infections, and pregnancy-associated pregnancy disorders, the reference category is the presence of these pathologies. Odds Ratios (OR) and 95% Confidence Intervals (CI) were calculated for each category.

## Data Availability

The data used to support the findings of this study are available upon request.

## Abbreviations

The following abbreviations are used in this manuscript:

AP	adolescent pregnancy
PROM	premature rupture of membranes
PPROM	preterm premature rupture of membranes
LGA	large-for-gestational-age
BMI	body mass index
kg	kilograms
AGA	according to gestational age
SGA	small for gestational age
FGR	fetal growth restriction
AC/EFW	abdominal circumference/estimated fetal weight
US-AEDF	umbilical artery absent end-diastolic flow
NICU	neonatal intensive care unit
C-sections	cesarean sections
HIV	Human Immunodeficiency Virus
LBW	low birth weight
HELLP Syndrome	Hemolysis, Elevated liver enzymes, Low platelet count
